# Genomic Profile and BRCA-1 Promoter Methylation Status in BRCA Mutated Ovarian Cancer: New Insights in Predictive Biomarkers of Olaparib Response

**DOI:** 10.3389/fonc.2019.01289

**Published:** 2019-11-29

**Authors:** Elisena Franzese, Sara Centonze, Anna Diana, Angela Lombardi, Francesca Carlino, Luigi Pio Guerrera, Ferdinando De Vita, Michele Caraglia, Sandro Pignata, Fortunato Ciardiello, Michele Orditura

**Affiliations:** ^1^Division of Medical Oncology, Department of Precision Medicine, School of Medicine, Luigi Vanvitelli University of Campania, Naples, Italy; ^2^Istituto Nazionale per lo Studio e la Cura dei Tumori “Fondazione G. Pascale”, IRCCS, Naples, Italy

**Keywords:** ovarian cancer, genomic profiles, BRCA 1/2 mutation carriers, promoter methylation, olaparib (Lynparza™)

## Abstract

**Objective:** We assessed the genomic profile of four representative BRCA-mutated ovarian cancer (OC) patients treated with olaparib to investigate the relationship between intratumor heterogeneity and response to olaparib treatment. The main aim is to identify possible predictive biomarkers of response to olaparib through the analysis of HRD or not HRD genes and the definition of BRCA1 promoter methylation status.

**Methods:** DNA, isolated from formalin-fixed, paraffin-embedded (FFPE) diagnostic OC tissues, was analyzed by FoundationOneCDx™. This assay detects alterations in a total panel of 324 genes, using the Illumina® HiSeq 4000 platform. Methylation analysis of the BRCA gene promoter was carried out by pyrosequencing with PyroMark Q24 platform (Qiagen), an *in vitro* nucleic acid sequence-based detection test based on pyrosequencing technology for quantitative measurements of methylation status.

**Results:** Case #1 and #2 were defined Long-term responders since they received olaparib for 27 and 36 months, respectively. These remarkable results could be explained, at least in part, by the presence of somatic IDH1 mutation in case #1 and PI3K and SOX2 amplification in the case #2. In case #3, the somatic NF1 mutation appeared to be related to the short duration of response. In the case #4, in which the patients is on olaparib from 1 year achieving a stable disease, a somatic mutation of BRCA1 was recorded. Moreover, in all cases, levels of BRCA1 promoter were strictly related to olaparib response.

**Conclusions:** Based on our experience, genomic analysis of tumor tissue at diagnosis might help to determine the future response to olaparib in advanced OC setting, revealing predictive biomarkers beyond BRCA 1-2 and HRD status.

## Introduction

Olaparib, niraparib, and rucaparib are the three poly (ADP-ribose) polymerase inhibitors (PARPis) approved by regulatory authorities for treatment of advanced ovarian cancer (OC) patients.

Olaparib was initially approved in December 2014 by the Food and Drug Administration (FDA) as monotherapy in patients with deleterious germ-line BRCA mutated (BRCAm) advanced OC treated with three or more prior lines of chemotherapy and by the European Medicines Agency (EMA) as maintenance treatment for platinum sensitive relapsed BRCAmOC (germline or somatic mutations). In August 2017, based on the encouraging long-term efficacy results of Study 19 (phase II trial) (1), the FDA expanded the label of olaparib as maintenance treatment for recurrent OC patients who are in complete or partial response following platinum-based chemotherapy, irrespective of their BRCAm status and the number of prior lines received. In May 2018, the EMA added the same indication. The results of SOLO1 phase III study (2) demonstrated that olaparib maintenance therapy reduced significantly the risk of disease progression or death in patients with newly diagnosed BRCAm OC. Therefore, in December 2018, the FDA approved olaparib also in this setting.

In 2017, niraparib and rucaparib have been licensed by the FDA as maintenance treatment of platinum-sensitive recurrent OC, regardless of BRCA mutational status, since they improved median progression-free survival (mPFS) in both BRCA wild type (wt) and BRCAm OC in ENGOT-OV16/NOVA and ARIEL3 phase III studies (3, 4), respectively.

In-depth analysis of olaparib data obtained from clinical trials clearly indicated BRCA 1/2 mutations as crucial predictive biomarkers. Genome wide loss of heterozigosity (LOH) and homologous recombination deficiency (HRD) are considered useful predictive markers of response to rucaparib and niraparib. HRD is a cellular weakness status resulting from disruption of genes involved in homologous recombination repair (HRR) of DNA double strand breaks. These alterations include genetic (germline or somatic) variants of BRCA, PALB2, ATM, FANCA, RAD51, CHEK2, BRIP1, or epigenetic inactivation (i.e., BRCA1 promoter hypermethylation) (5). LOH is a signature of HRD that is an indirect measure of genomic instability (6). In ENGOT-OV16/NOVA trial, HRD was assessed using an assay (*myChoice HRD test*) which yields a score based on LOH, telomeric allelic imbalance, and large-scale state transition. Furthermore, the beneficial effects of niraparib in HRD negative OC population has opened the way for new research to identify novel predictive biomarkers to PARP inhibition, beyond BRCA and HRD. Indeed, the expression of SLFN11, loss of RB1, TP53, MYC amplification, and high levels of E-cadherin recently emerged as promising predictive biomarkers (7, 8).

In this study, we investigated the genomic profile of BRCAm OC patients to assess the relationship between intratumor heterogeneity and response to olaparib treatment through analysis of mutations of HRD or not HRD genes and definition of BRCA1 promoter methylation status.

## Methods

We selected four representative BRCAm OC patients who achieved different responses to olaparib therapy, ranging from complete response (CR) to progressive disease (PD). Patients were classified as long-term (LT) responders if mPFS was >2 years and short-term (ST) responders if it was ≤3 month. Efficacy data analysis was updated in June 2019.

DNA, isolated from formalin-fixed, paraffin-embedded (FFPE) diagnostic ovarian tumor tissues, was analyzed by FoundationOneCDx™. This assay detects alterations in a total panel of 324 genes. Using the Illumina® HiSeq 4000 platform, hybrid capture-selected libraries were sequenced to high uniform depth (targeting >500X median coverage with >99% of exons at coverage >100X). Sequence data were then processed using a customized analysis pipeline designed to detect all classes of genomic alterations, including base substitutions, indels, selected genomic rearrangements (e.g., gene fusions), and copy number alterations (amplifications and homozygous gene deletions). The threshold used in FoundationOneCDx for identifying a copy number amplification was 4 for ERBB2 and 6 for all other genes (9). Additionally, genomic signatures including microsatellite instability (MSI) and tumor mutational burden (TMB) were reported.

Furthermore, methylation analysis of the BRCA gene promoter was carried out by pyrosequencing. PyroMark Q24 platform (Qiagen), an *in vitro* nucleic acid sequence-based detection test based on pyrosequencing technology for quantitative measurements of methylation status in exon 1 of the human BRCA1 gene in genomic DNA derived from human tissue sample, was used. For methylation analysis, specific primers for CpG island of BRCA1 gene for two target regions were designed (Table 1).

**Table 1:** Primers for F1 (forward), R1 (reverse), S1 (sequencing primers) target region 1 and primers for F2 (forward), R2 (reverse), S2 (sequencing primers) target region 2.

**Primer**	**Id**		**Sequence**	**Nt**	**Tm, ^**°**^C**	**%GC**
					**Score: 61**	
		**Primer set 1**			**Quality: Medium**	
⇀PCR	F1		GGGGTAGATTGGGTGGTTAATTT	23	60.8	43.5
↽PCR	R1		CCAATACCCCAAAACATCACTT	22	59.2	40.9
→ Sequencing	S1		TTTGAGAGGTTGTTGTTTA	19	44.1	31.6
					**Score: 64**	
		**Primer set 2**			**Quality: Medium**	
⇀PCR	F2		GGGTAGATTGGGTGGTTAATT	21	59	42.9
↽PCR	R2		CCAAAACATCACTTAAACCCCCTAT	25	62.2	40
←Sequencing	S2		ATTATCTAAAAAACCCCACAA	21	44.7	28.6

QIAmpEpitect FFPE Lysis Kit was used for extraction of human DNA from FFPE tumor samples. For bisulfite conversion, the Epitect Bisulfite Kit from QIAgenis was used. Five microliter bisulfite-converted template DNA (40 ng of genomic DNA) was added. A sample with methylated control DNA was included as a positive control for PCR and sequencing reactions. Afterwards, immobilization of PCR products to streptavidin sepharose high performance beads was carried out. The single-stranded DNA was then prepared and annealing of the sequencing primer to the template, prior to pyrosequencing analysis on the PyroMArk Q24, was carried out. This method was intended to quantitatively measure methylation in two CpG sites in exon 1 of the human BRCA1 gene.

Bisulfite-converted genomic DNA was amplified by PCR and sequenced through the defined region in the forward direction. Sequences surrounding the defined positions served as normalization and reference peaks for quantification and quality assessment of the analysis.

After PCR, the amplicons were immobilized on streptavidin sepharose high performance beads (GE HealthCare). Single-stranded DNA was prepared, and the sequencing primer annealed to the DNA. The samples were then analyzed on the PyroMark Q24 System using a specific assay setup file and a specific run file.

Methylated control DNA was included as a positive control (PC) for PCR and sequencing reactions. In addition, a negative control (not methylated DNA-NTC) was included in every PCR setup (Table 1).

The methylation level of each CpG site was estimated by the proportion of C (%) in each region. The mean of the unmethylated (UM) sample was 4.3% in region 1 and 6.6% in region 2; therefore, the samples with the same % of methylation were not methylated.

Evaluation of low levels of methylation or unmethylation is difficult to analyze and depends on the correct use of the unmethylated control in the analysis. For method comparisons, unmethylated or methylated status were assigned to the pyrosequencing analysis results using 7% units as mean methylation of CpG. All patients gave written informed consent to participate in this study and for the publication of any potentially identifiable data included in the article.

## Results

All patients had diagnosis of high grade serous ovarian cancer (HGSOC), three harbored a germinal BRCA1 mutation (gBRCA1) and one had a somatic mutation (sBRCA1). Case #1 and #2 were defined LT responders since they received olaparib for 27 and 36 months, with CR and PR, respectively. Case #3 was considered ST responder and experienced PD after 4 months of treatment. The somatic BRCA1m patient (case #4) is still continuing olaparib after 1 year, with stabilization of disease (SD).

In detail:

**Case #1:** A 50-year-old Polish patient underwent hysteroannessiectomy, appendectomy, and omentectomy in October 2012 for bilateral HGSOC, IIIC FIGO stage, gBRCA1 mutated. She reported positive family history for breast carcinoma. From January to May 2013, six cycles of carboplatin and paclitaxel were administered, as first line therapy. From October 2015 to June 2016, due to retroperitoneal relapse, she received a second-line chemotherapy with carboplatin, paclitaxel and bevacizumab, obtaining a PR. In September 2016, she underwent debulking surgery with microscopic residual tumor (RT1). From November 2016 to February 2017, she was administered four cycles of cisplatin, with further reduction of residual tumor burden. In March 2017, the patient started maintenance therapy with olaparib capsules at a dose of 400 mg twice daily. After 3 cycles, she obtained a CR and the treatment is still ongoing.

In case #1, the molecular characterization revealed:

- a *BRCA1 c.181T*>*G (p.Cys61Gly*) missense mutation located within exon 5, consisting of a T>G substitution at position 181, resulting in a substitution of cysteine with glycine at codon 61. This mutation falls in the RING finger domain of BRCA1 protein and is a founder mutation in the Polish population;
- a *c.602T*>*A (p.Lys201Ter)* non-sense mutation of TP53 gene, located in exon 6, which results in the introduction of a stop codon at aminoacid position 201. This alteration affects the DNA binding domain;
- a *IDH1 c.145C*>*T (p.Arg49Cys)* missense mutation, causing a substitution of arginine to cysteine at codon 49, which does not lie within any known functional domains but results in decreased accumulation of IDH1 protein in cell culture.

Quantitative measurements of methylation status in exon 1 of BRCA1 gene showed that mean and median methylation levels of region 1 were 39.25 and 39%, respectively, and mean and median of methylation levels of region 2 were 17.75 and 17.5%, respectively.

**Case #2**: A 63-year-old patient with right ovary HGSOC, IIIA FIGO stage, gBRCA1 mutated, without family history for breast or OC, was subjected to radical hysteroannessiectomy and pelvic lymphadenectomy in February 2013. From April to July 2013, the patient received 6 cycles of carboplatin and paclitaxel as adjuvant chemotherapy. In May 2014, because of left inguinal lymph node involvement (progressive disease), the patient was treated with carboplatin, paclitaxel, and bevacizumab for six cycles as first-line treatment, followed by bevacizumab maintenance until June 2015. The best response was SD. From January 2016 to June 2016, for intercaval-aortic lymph node relapse, the patient received second-line chemotherapy with cisplatin and gemcitabine for 4 cycles, obtaining a PR; she subsequently started olaparib maintenance. The treatment is still ongoing, and the best response obtained is SD.

In case #2, molecular characterization revealed:

- a *c.117_118delTG (p.Cys39Terfs)* mutation of BRCA1 gene, located within exon 3, consisting of a TG deletion at position c.117_118, yielding a reading frame shift at codon 39, with consequent premature termination at codon 40;
- a *c.733G*>*A (p.Gly245Ser)* missense mutation of the TP53 gene located in exon 7, consisting of a G>A substitution at position 733, leading to a substitution of glycine with serine at codon 245. This mutation impacts DNA binding and transcriptional activation;
- a *SMARCA4* frameshift mutation (p.Phe1276fs^*^15). This gene encodes the protein BRG1, an ATP-dependent helicase that regulates gene transcription through chromatin remodeling. SMARCA4 mutation has been reported in 1.3% of serous OC, while it represents a pathogenic molecular feature of small cell carcinoma of the ovary, hypercalcemic type (SCCOHT), an aggressive form affecting children and young women;
- Amplification of the following genes:
  - *PIK3CA*, which encodes p110-alpha, the catalytic subunit of phosphatidylinositol3-kinase (PI3K). Amplification of this gene constitutively upregulates the PI3K/AKT/mTOR pathway, which has been associated with resistance to standard cancer therapies;
  - *SOX2*, which encodes a transcription factor described as a lineage survival oncogene. SOX2 amplification or overexpression leads to activation of the PI3K/AKT/mTOR pathway;
  - *LYN*, which encodes an SRC family intracellular membrane-associated tyrosine protein kinase, involved in the activation of the PI3K/AKT/mTOR pathway;
  - *PRKCI*, which encodes protein kinase C iota (PKCi). PKCi activation has been reported to promote hedgehog signaling as well as RAS signaling. It requires the RAC1-MEK-ERK pathway for tumorigenesis;
  - *TERC*, the human telomerase RNA gene (hTERC), which encodes the RNA component of the telomerase enzyme. Telomerase is an RNA polymerase that maintains telomeric DNA and plays a role in senescence and oncogenesis.

Quantitative measurements of methylation status in exon 1 of BRCA1 promoter methylation showed that mean and median methylation levels of region 1 were 17.25 and 17%, respectively, while mean and median methylation levels of region 2 were 3.3 and 3.5%, respectively.

**Case #3:** A 36-year-old patient with bilateral HGSOC, stage IV for liver metastasis, gBRCA1 mutated, with no family history for breast carcinoma and OC, underwent bilateral hysteroannessiectomy in April 2016. In May 2016, she started first-line chemotherapy with carboplatin, paclitaxel, and bevacizumab for 6 cycles, followed by bevacizumab maintenance until November 2016. Due to lymph node and peritoneal progression, a second-line treatment with carboplatin single agent for 4 cycles was started. After achieving a PR, the patient started treatment with olaparib in January 2018. In May 2018, CT scan assessment demonstrated SD. In June 2018, acute renal failure occurred, and CT scan revealed retro-peritoneal, pleural, and lymph node progression. In August 2018, a third-line chemotherapy with carboplatin and paclitaxel was started. The patient died in December 2018.

The molecular characterization revealed:

- a c.3756_3759delGTCT (p.Ser1253Argfs^*^10) mutation of the BRCA1 gene, located within exon 11 and consisting of a GTCT deletion at position c.3756_3759, resulting in a reading frame shift at codon 1253, with downstream premature termination at codon 1263;
- a *c.425*+*2T*>*C (IVS4*+*2T*>*C)* intronic variation of the BRCA2 gene. This substitution was considered likely pathogenic because it was predicted to affect or create spice donor or splice acceptor sites, as reported in Invitae Variant Classification Sherloc (version n. 09022015);
- a *c.1025G*>*C (p. Arg342Pro)* missense mutation of the TP53 gene, localized within exon 10 and consisting of a substitution of arginine with proline at codon 342. This genomic event has been recently related to compromised tetramerization and transcriptional ability of p53;
- a frameshift NF1 mutation (p.E2490fs^*^11), related to a single nucleotide sequence determining a guanine with adenine substitution. This alteration, albeit not altering the GTPase domain, is involved in carcinogenesis with a still unknown mechanism.

**Figure 1:**
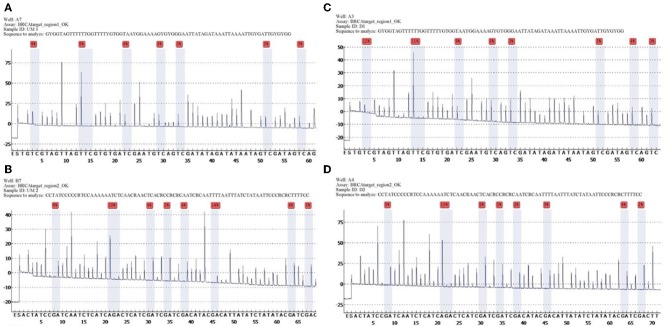
Unmethylated control and histograms of unmethylated case 3. BRCA1 promoter methylation level in ovarian cancers quantified by pyrosequencing. The percentages (red) are the proportion of C at each CpG site after bisulfite conversion, and the methylation level of each CpG site is estimated by the proportion of C (%). An overall BRCA1 promoter methylation level is calculated as the average of the proportions of C (%) at 8 CpG sites. Representative pyrograms: **(A)** pyrogram of a tumor DNA showing heterogeneous levels of methylation at TC sites in the CpG island of the BRCA1 promoter. The y-axis represents the signal intensity in arbitrary units, the x-axis shows the dispensation sequence. UM Region 1 **(A)**, UM Region 2 **(B)**, case 3 Region 1 **(C)**, case 3 Region 2 **(D)**.

Quantitative measurements of methylation status in exon 1 of BRCA1 gene showed that mean and median methylation levels of region 1 were 6.6 and 5.5%, respectively, whereas mean and median methylation levels of region 2 were 3.6 and 3%, respectively (Figure 1).

**Case #4:** A 60-year-old patient with HGSOC, stage IIIC FIGO, germinal BRCA1/2 wt, underwent bilateral hysteroannessiectomy and systematic pelvic lymphadenectomy in April 2015. She received first-line chemotherapy with carboplatin, paclitaxel and bevacizumab for 6 cycles, completing bevacizumab maintenance in October 2016. Due to abdominal relapse, she underwent right hemicolectomy in October 2017; tumor sample analysis revealed somatic BRCA1 mutation. From November to May 2018, she received 6 cycles of cisplatin, achieving a PR. Thus, maintenance therapy with olaparib was started in June 2018. The treatment is still ongoing, and SD has been the best response obtained.

The molecular characterization revealed:

- a c.4595_4596insCT (p.Asp1533Leufs^*^16) mutation of BRCA1 gene, localized within exon 15 and consisting of a CT insertion at position c.4595_4596, resulting in a reading frame shift at codon 1533, with downstream premature termination of codon 16. This alteration falls between BRCT e coiled-coil domains, leading to a function loss of BRCA1 protein;
- a *c.488A*>*G (p.Tyr163Cys)* missense mutation of TP53 gene, located in exon 5 and causing a substitution of tyrosine with cysteine at codon 163. This alteration lies within the DNA binding domain of Tp53 protein, resulting in decreased transactivation of Tp53 target genes, increased cellular growth rate, and failure to induce apoptosis in cell culture;
- *FGF12* equivocal amplification. Mutations in FGF12, a member of the fibroblast growth factor (FGF) family, are rare (<1%) in cancer. FGF12 expression has been found to be increased in the diffuse type of gastric cancer and downregulated in breast cancer samples from patients with a pathological complete response (pCR) following chemotherapy.

Quantitative measurements of methylation status in exon 1 of BRCA1 gene showed that mean and median methylation levels of region 1 were 18.8 and 18%, respectively, while mean and median methylation levels of region 2 were 6.8 and 6%, respectively.

The list of identified mutations and the percentages of methylation in each sample are reported in Tables 2, 3; the unmethylated control and histograms of the unmethylated case are reported in Figure 1.

**Table 2:**
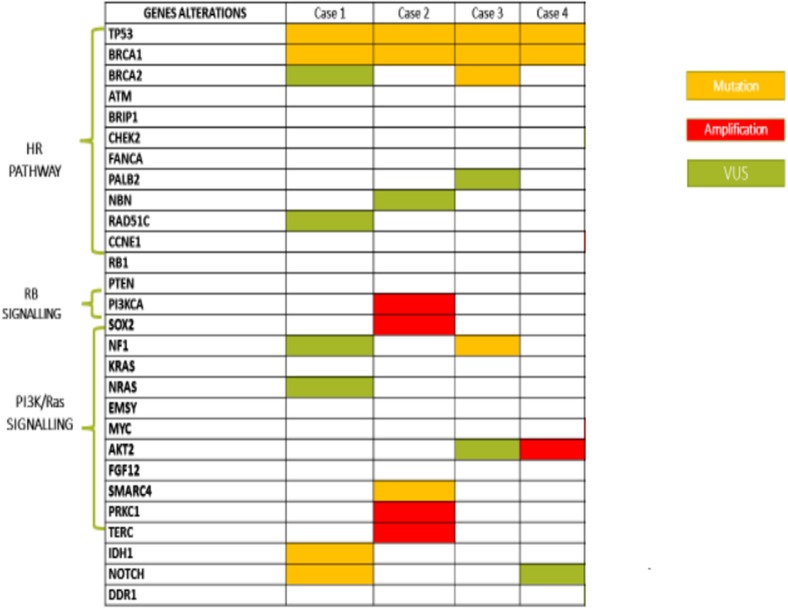
List of mutations identified.

**Table 3:** Percentages of methylation in each sample with means and medians in Region 1 and Region 2.

	**Case #1**	**Case #2**	**Case #3**	**Case #4**
	**Complete response/Long term responder (27 months)**	**Partial response/Long term responder (36 months)**	**Progression disease/Short term responder (4 months)**	**Stability disease/sBRCAm (12 months)**
Mean Region 1	39.25% (methylated)	17.25% (low methylated)	6.6% (unmethylated)	18.8% (low methylated)
Mean Region 2	17.75% (methylated)	3.3% (unmethylated)	3.6% (unmethylated)	6.8% (unmethylated)
Median Region 1	39% (methylated)	17% (low methylated)	5.5% (unmethylated)	18% (low methylated)
Median Region 2	17.5% (methylated)	3.5% (unmethylated)	3% (unmethylated)	6% (unmethylated)

*In Red, highly methylated samples; In black, unmethylated samples; In blue, low methylation samples. sBRCAm; somatic BRCA mutated*.

## Discussion

The first clinical and molecular olaparib response characterization was carried out in 51 OC patients, recruited in study 19 and 41 (10). Patients were classified as LT responders if mPFS was >2 years and ST responders if it was <3 months. The molecular profile was defined by assessment of germline and somatic BRCA1/2 mutations with determination of three biomarkers of HRD score and by evaluation of BRCA1 methylation. Mutational profile by the Foundation Medicine T5 panel detected genetic mutations and amplifications in genes encoding proteins involved in DNA repair and damage response, regulation of cell cycle, apoptosis, and MAPK/PI3K signaling. In order to identify predictive biomarkers of response for BRCAwt patients who had received benefit from olaparib treatment, another exploratory analysis of the Study 19 was carried out (11). Patients were characterized based on HR status and subdivided into BRCA wt/HR mutated and BRCA wt/HR wt. Olaparib therapy seemed to be associated with a longer PFS benefit in HR-mutated patients without a BRCA mutation with respect to patients with no detectable BRCA or HR mutation. Moreover, from this analysis, silencing of BRCA1 through promoter methylation was not shown to result in improvement in response rates to platinum-based chemotherapy, sequential chemotherapy, and maintenance olaparib therapy. This observation was previously suggested by The Cancer Genome Atlas (TCGA), but other studies failed to demonstrate a significant improvement in overall survival upon stratification by BRCA1 methylation status (12–14).

However, the ARIEL2 Part 1 trial demonstrated that BRCA1 promoter methylation increased LOH levels. Recently, zygosity status (homozygous or hemizygous vs. heterozygous) of BRCA1 promoter methylation has been shown to affect rucaparib and platinum response (15). Homozygosity defines the methylation status in which unmethylated alleles are absent, regardless of BRCA1 copy number, and predicts rucaparib response, whilst heterozygous methylation is associated with resistance. Furthermore, methylation loss can occur after exposure to chemotherapy, allowing for rapid development of drug resistance.

Our experience suggests that genomic characterization of BRCA mutated OC patients allows identification of molecular pathway alterations that mostly interfere with olaparib response. Consistent with these findings, we also observed that BRCA1 promoter methylation status influenced olaparib response.

In case #1, complete response and long-term olaparib treatment could be explained, at least in part, by somatic IDH1 mutation. The normal function of IDH enzymes is to catalyze the conversion of isocitrate to α-ketoglutarate (αKG) in the citric acid cycle. When IDH1/2 heterozygous mutations occur, encoded enzymes gain a neomorphic activity so as to convert α-KG to an oncometabolite, (R)-2HG (16, 17). It exhibits pleiotropic effects on cell biology, including direct inhibition of αKG-dependent dioxygenases, in particular KDM4A and KDM4B, involved in HRR. IDH1 mutant tumor cells, resulting in an HRD status, are known to be sensitive to olaparib activity, approaching a 50-fold difference compared to IDH1-wt cells (18).

Moreover, in this case, high levels of BRCA1 promoter gene methylation correlated with olaparib complete and long-term response.

In case #2, SMARCA4 mutation was found. The encoded protein, BRG1, promotes the repair of DNA double-strand breaks by facilitating the replacement of RPA with RAD51. Loss of BRG1 results in failure of RAD51 loading onto ssDNA, abnormal HRR, and enhanced DSB-induced lethality. Hence, SMARCA4 loss may also enhance tumor sensitivity to PARPis (19).

However, SD and LT olaparib treatment could be explained by PI3K and SOX2 amplification. Aberrant activation of PI3K pathway is known to maintain HR steady state (20), and recent studies have shown that PI3K blockade with BKM120 results in impaired DNA HRR and sensitivity to olaparib in TNBCs with proficient or deficient BRCA genes (21). Furthermore, the effect of combined use of PI3K and PARPis on OC cell lines with mutant PIK3CA has also been explored (22). This combination has been shown to synergize in inhibiting proliferation, survival, and invasion in most of OC cell lines harboring PIK3CA mutations. Combined treatment resulted in an exacerbated DNA damage response, decreased BRCA1/2 expression, and more substantially reduced AKT/mTOR signaling when compared to single agent. For this patient, further data support the benefit that could derive from PI3K/PARP combination, particularly, SOX2 gene amplification leading to PI3K pathway upregulation (23). In fact, PI3K and AKT inhibitors, in several preclinical studies, were demonstrated to be able to reduce SOX2-driven growth, viability, migration, tumorigenicity, and drug resistance of cancer cells (24). A recent study showed that SOX2 overexpression was found in a proportion of women with BRCA1 and BRCA2 mutations who underwent prophylactic salpingo-oophorectomy, and in the majority of patients with HGSOCs, irrespective of tumor stage (25). The authors proposed SOX2 overexpression in fallopian epithelial tube as a biomarker for detecting disease at a premalignant stage. Further, low methylation levels of BRCA1 promoter gene could explain an attenuated olaparib clinical response.

In case #3, the somatic NF1 mutation appeared to be related to the short duration of response and the rapid progression to subsequent platinum therapy. There are few clear prognostic data on the influence of NF1 loss in HGSC patient survival, while clinical and preclinical studies have documented resistance to platinum-based chemotherapy (26). Recently, the effects of Tp53 and NF1 mutations on sensitivity to the PARP inhibitor rucaparib and to platinum chemotherapy was investigated on OC cells *in vitro* and both *in vitro* and *in vivo*, respectively (27). Tumors with double Tp53 and NF1 mutations had an increased rate of intra-tumoral growth, reduced rucaparib sensitivity, and worst survival following platinum treatment compared to those with Trp53 mutation alone. Based on clinical and strong preclinical evidence, NF1 inactivation may predict sensitivity to MEK inhibitors (28). In this case, the short duration of response and the rapid progression could be attributed to the unmethylated BRCA1 promoter gene.

In case #4, a somatic mutation of BRCA1 was recorded. A retrospective molecular analysis of Study 19 on available tumor tissues supports its predictive value (29). NGS identified somatic mutations absent from germline testing in 10% (20/209) of patients. Somatic mutations had >80% biallelic inactivation frequency and were predominantly clonal, suggesting that BRCA1/2 loss occurs early in the development of these cancers. Clinical outcomes between placebo- and olaparib-treated patients with somatic BRCA1/2 mutations were similar to those with germline BRCA1/2 mutations. Moreover, low levels of BRCA1 promoter gene methylation correlated with olaparib clinical response.

All patients exhibited low TMB levels. This is a measure of the number of somatic protein-coding base substitution and insertion/deletion mutations occurring in a tumor specimen. Based on emerging clinical evidence, increased TMB may be associated with greater sensitivity to immunotherapeutic agents, including anti-CTLA-4, anti-PD-L1, and anti-PD-1 therapies (30). However, BRCA mutated tumors had increased CD3+ and CD8+ immune infiltrates and expression of PD-1/PD-L1 compared with HR proficient tumors (31). Dai et al. demonstrated that TMB failed to reflect the immunogenicity of HGSOC, since only a slight correlation with cytolytic immune response and immune cells infiltration of HGSOC was found (32). These results suggest that TMB might not be a valid predictive biomarker for HGSOC immunotherapies. Furthermore, the authors identified higher levels of 10 immunological factors in BRCA1-mutated tumors when compared to wild-type ones, regardless of TMB, since no differences between the BRCAwt group and the BRCAm group were observed. Moreover, the typical immunoreactivity profile exhibited by HRD tumors would depend only on the neo-antigen load from the degradation process of damaged DNA.

All patients analyzed harbored p53 somatic mutations. TP53 alterations have been reported in 29–80% of ovarian tumors, with a higher incidence in high-grade pelvic (primary ovarian, tubal, or peritoneal) serous carcinoma (91–97%) (33). TP53 alterations have also been reported in serous tubal intraepithelial carcinomas (STICs) of the fallopian tube, which are thought to be precursor lesions of HGSOC (34). Missense mutations leading to TP53 inactivation may also be sensitive to therapies that reactivate mutant p53, like APR-246 (35). In a phase 1b trial in patients with p53-positive HGSOC, APR-246 combined with carboplatin and pegylated liposomal doxorubicin achieved a 52% (11/21) response rate and a 100% disease control rate (36).

## Conclusions

Olaparib is the first approved PARP inhibitor and has already changed treatment paradigms for subgroups of OC patients with mutated BRCA. To date, candidate biomarker analyses beyond BRCA genes have been descriptive but not interpretative. Therefore, while waiting for reliable biomarkers for LT responders to olaparib, the results of observational studies may be informative. OLALA, an ongoing observational study of long-term responders to olaparib, has collected samples from patients (alive or deceased) involved in several clinical trials using olaparib as investigational drug. Outcomes will identify signature of PARP response and resistance in different tumor sites. Moreover, preclinical and clinical studies are currently testing olaparib combination therapies with immune checkpoint inhibitors, antiangiogenics, or other agents, in order to confer HR deficiency in HR-proficient tumors, so as to sensitize them to PARP inhibition. Based on our experience, genomic analysis of tumor tissue at diagnosis may help characterize the future response to olaparib in the relapse setting. In the next future, analysis of circulating free DNA (cfDNA) or DNA from circulating tumor cells (ctDNA) will enhance the genomic potential predictive role just at diagnosis, without neglecting the typical spatial-temporal heterogeneity of OC.

## Data Availability Statement

This manuscript contains previously unpublished data. The datasets analyzed for this study can be found in the GenBank.

## Ethics Statement

Ethical review and approval was not required for the study on human participants in accordance with the local legislation and institutional requirements. The patients/participants provided their written informed consent to participate in this study. Written informed consent was obtained from the individual(s) for the publication of any potentially identifiable images or data included in this article. All patients gave written informed consent to participate in this study and for the publication of any potentially identifiable 91 data included in the article.

## Author Contributions

EF: conceptualization, methodology, and writing original draft. SC: data creation and writing-original draft. AD: validation and review and editing. AL and MC: investigation, resources, and methodology. FCa and LG: software and resources. FD, SP, and FCi: supervision. MO: data creation, supervision, and writing-original draft.

### Conflict of Interest

The authors declare that the research was conducted in the absence of any commercial or financial relationships that could be construed as a potential conflict of interest.
